# Supplementary material for: An artificial cell capable of signal transduction mediated by ADRB2 for the regulation of glycogenolysis
Source: Nat Commun. 2026 Jan 16;17:1795. doi: 10.1038/s41467-026-68503-3 (PMC12916806; doi:10.1038/s41467-026-68503-3)
Supplement: Supplementary file 1 — Supplementary information [file 41467_2026_68503_MOESM1_ESM.pdf]

Supplementary Information for

An artificial cell capable of signal transduction mediated by ADRB2 for the regulation of glycogenolysis

Yanhao Liu[1, 2 3], Wan Zhao[1, 2 3], Yingming Zhao[1, 2 3], Xiangxiang Zhang[1, 2 3], Jingjing Zhao[1, 2 3], Shubin Li[1, 2 3]*, Yongshuo Ren[1, 2 3], Xiaojun Han[1, 2 3]*

[1]State Key Laboratory of Urban-rural Water Resource and Environment, School of Chemistry and Chemical Engineering, Harbin Institute of Technology, Harbin 150001, China.
[2]MIIT Key Laboratory of Critical Materials Technology for New Energy Conversion and Storage, School of Chemistry and Chemical Engineering, Harbin Institute of Technology, Harbin 150001, China.
[3]Heilongjiang Provincial Joint Laboratory of Molecular Science (International Cooperation), School of Chemistry and Chemical Engineering, Harbin Institute of Technology, Harbin 150001, China.
*Corresponding author. Email: hanxiaojun@hit.edu.cn
lishubin@hit.edu.cn

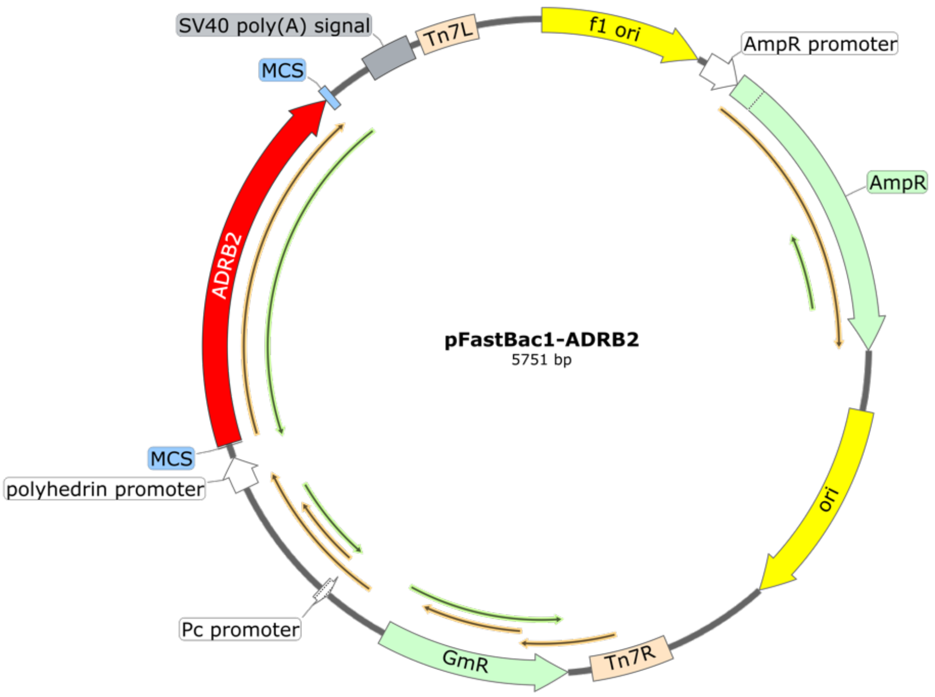

Supplementary Fig. 1. Plasmid map of pFastBac1-ADRB2.

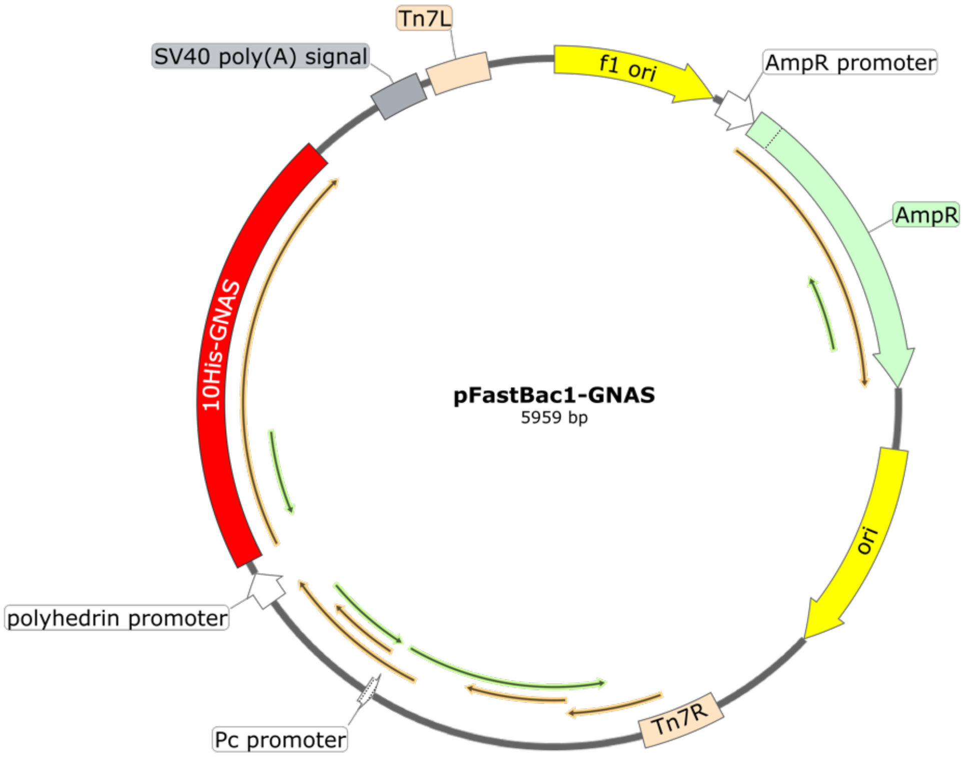

Supplementary Fig. 2. Plasmid map of pFastBac1-Gsα.

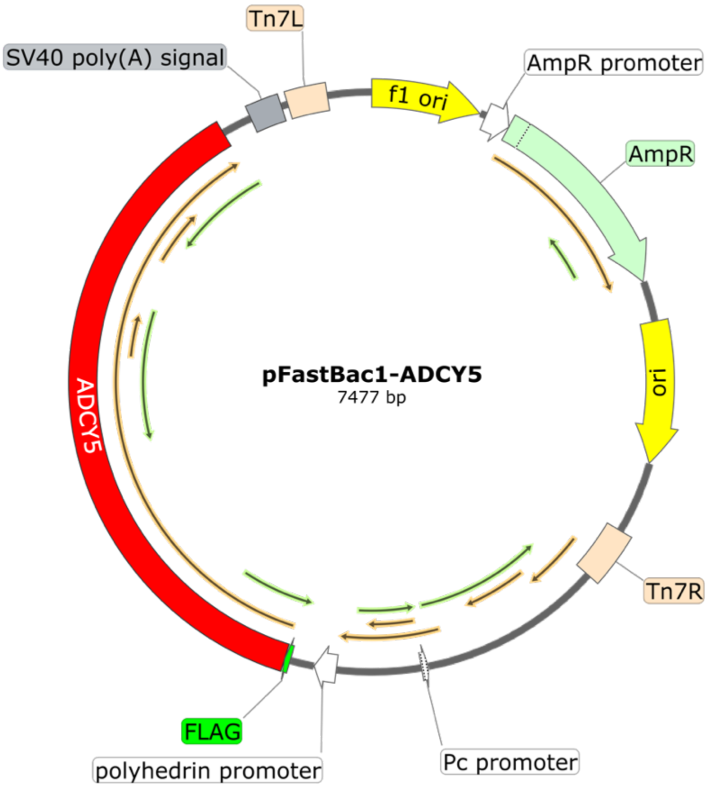

Supplementary Fig. 3. Plasmid map of pFastBac1-ADCY5.

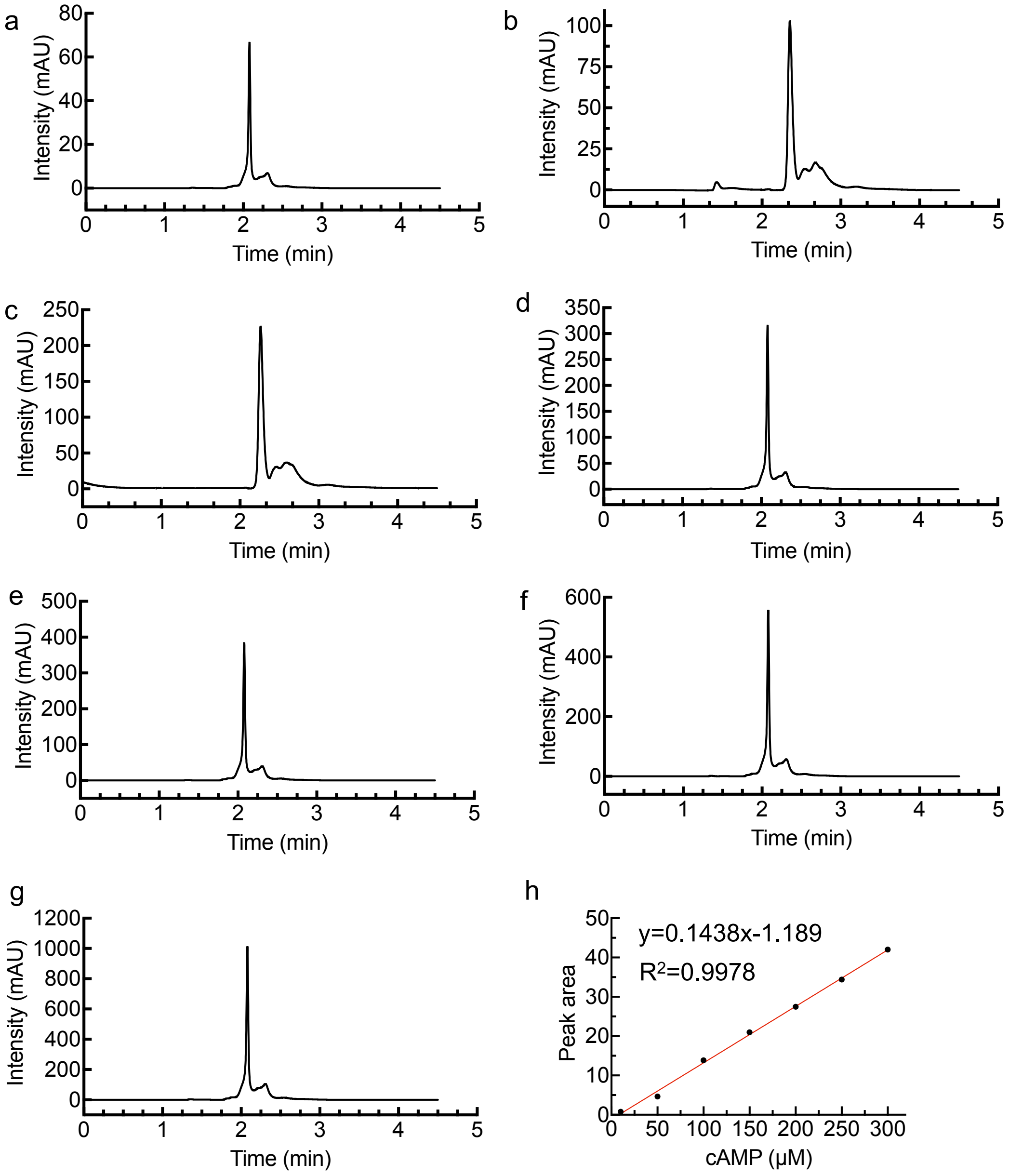

Supplementary Fig. 4. Representative high-performance liquid chromatography (HPLC) chromatograms of cAMP at concentrations of 10 μM (a), 50 μM (b), 100 μM (c), 150 μM (d), 200 μM (e), 250 μM (f), and 300 μM (g). (h) The calibration curve of cAMP detected by HPLC. Source data are provided as a Source Data file.

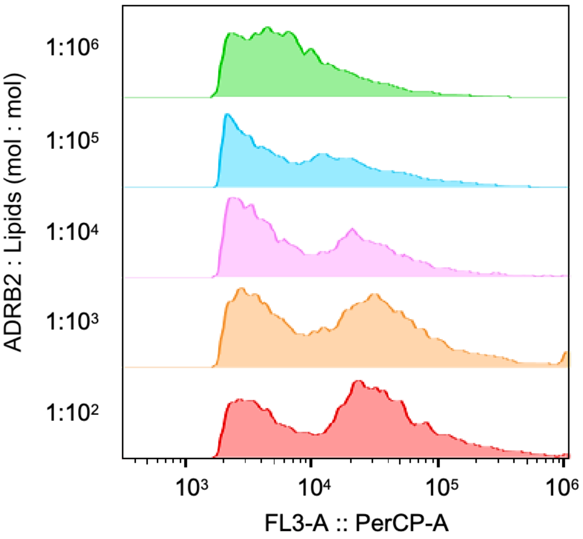

Supplementary Fig. 5. The flow cytometry histogram of Cy5-ADRB2-GUVs with varied ADRB2 to lipids molar ratio from $1{:}10^6$ to $1{:}10^2$.

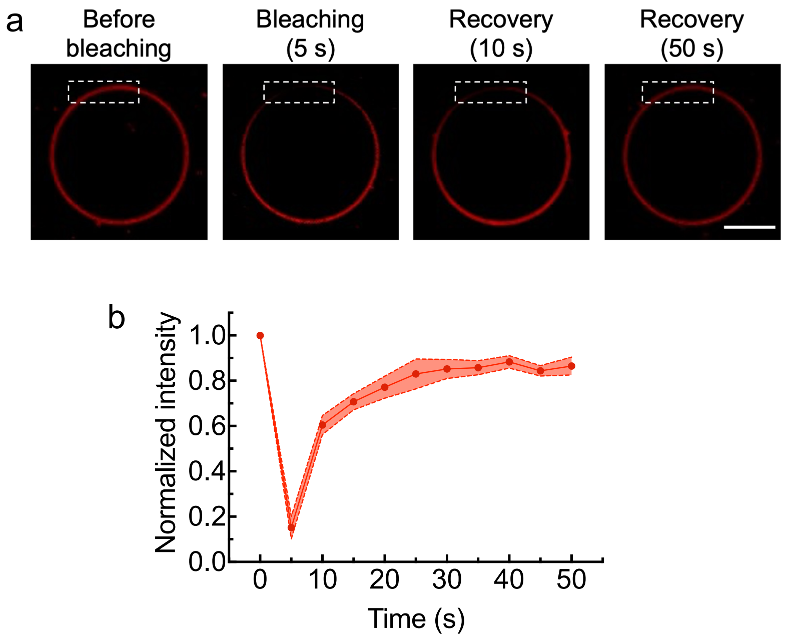

Supplementary Fig. 6. (a) Confocal fluorescence images of a single GUV membrane reconstituted with cy5-ADRB2 before photobleaching (left image), and after recovery (the other three images) at different time intervals. The photobleached area is indicated by the white box. (b) The corresponding fluorescence intensity of GUV membrane in the dashed white box in (a). The data were expressed as mean ± standard deviation (SD), n = 3 independent replicates. Source data are provided as a Source Data file.

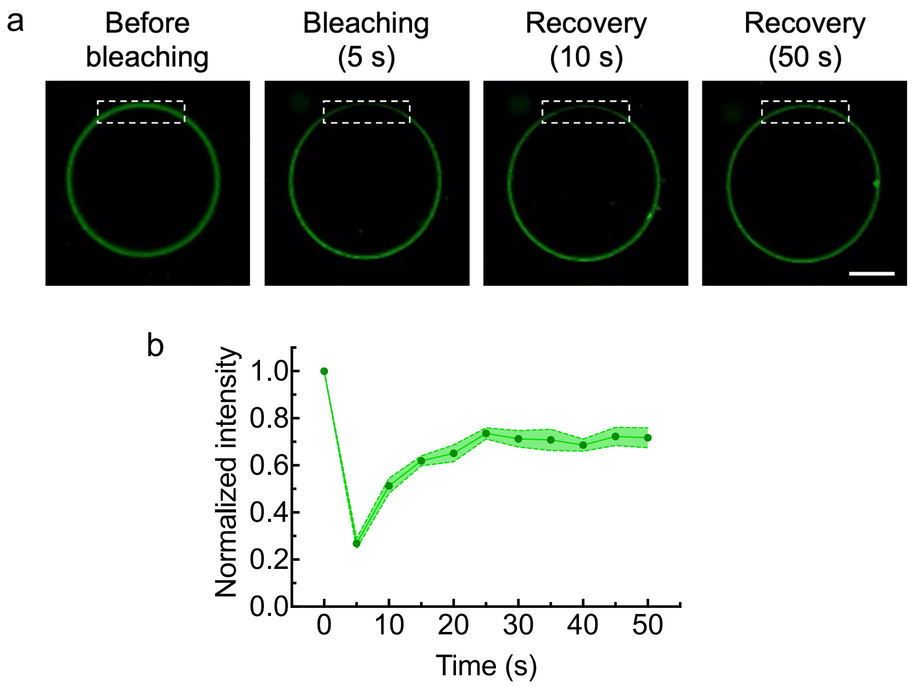

Supplementary Fig. 7. (a) Confocal fluorescence images of a single GUV membrane reconstituted with FITC-ADCY5 before photobleaching (left image), and after recovery (the other three images) at different time intervals. The photobleached area is indicated by the white box. (b) The corresponding fluorescence intensity of GUV membrane in the dashed white box in (a). The data were expressed as mean ± standard deviation (SD), n = 3 independent replicates. Source data are provided as a Source Data file.

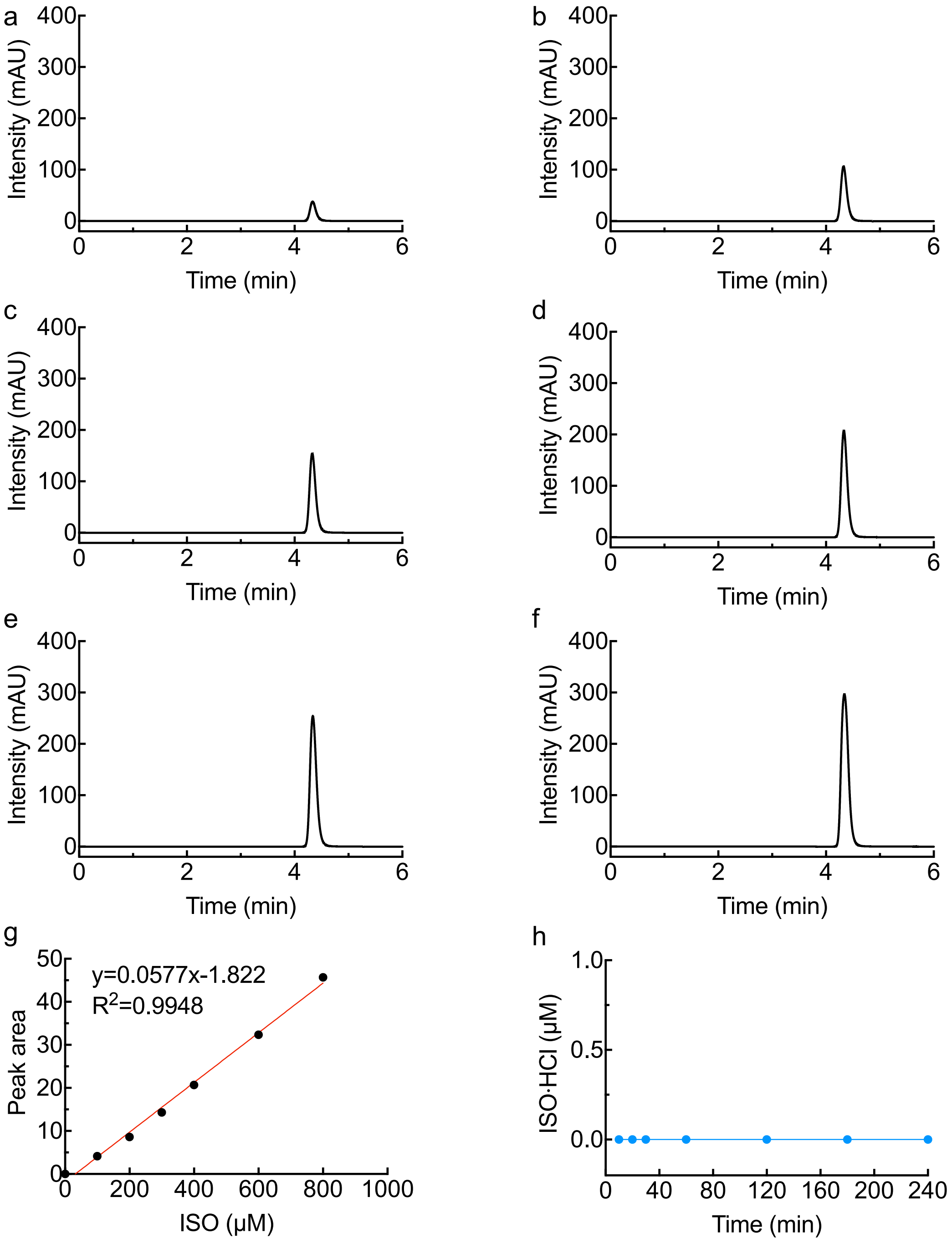

Supplementary Fig. 8. Representative high-performance liquid chromatography (HPLC) at concentrations of 100 μM (a), 200 μM (b), 300 μM (c), 400 μM (d), 600 μM (e), and 800 μM (f). (g) The calibration curve of ISO detected by HPLC. (h) The monitor of concentration of ISO outside GUVs as function of time from 10 to 240 min. Source data are provided as a Source Data file.

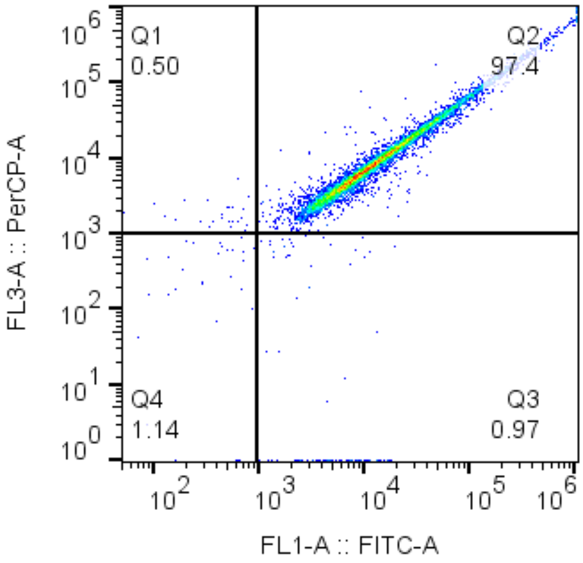

Supplementary Fig. 9. Two-dimensional flow cytometry scatter plots of artificial cells reconstituted with both ADRB2-Gsα complexes and ADCY5 with Y-axis of PerCP-A and X-axis of FITC-A.

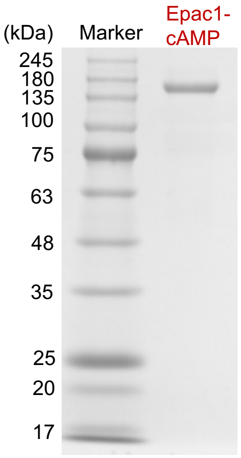

Supplementary Fig. 10. SDS-PAGE image of purified Epac1-cAMP. n = 3 independent replicates. Source data are provided as a Source Data file.

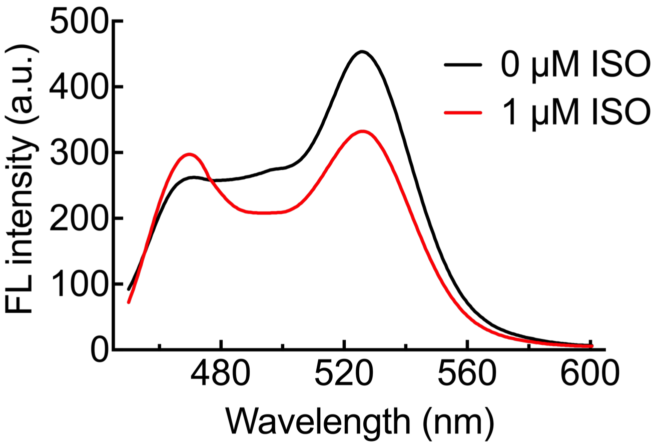

Supplementary Fig. 11. Fluorescence spectra of the Epac1-cAMP probe with the addition of 0 μM (black curve) and 1.0 μM (red curve) ISO. Source data are provided as a Source Data file.

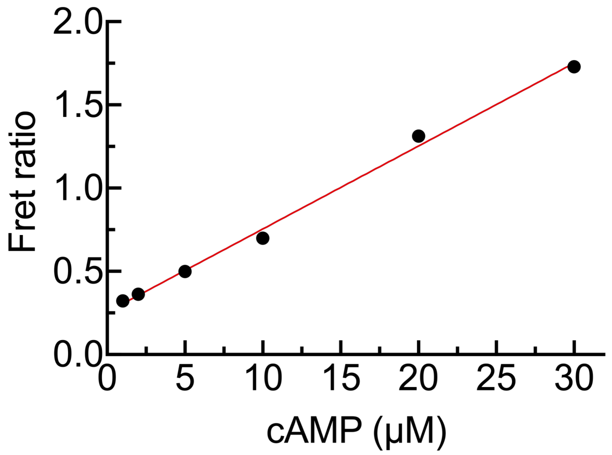

Supplementary Fig. 12. The calibration curve of cAMP in artificial cell measured by fluorescence spectroscopy. Source data are provided as a Source Data file.

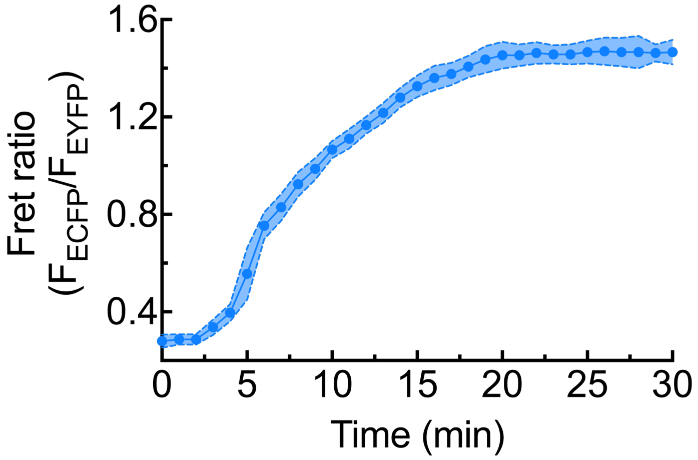

Supplementary Fig. 13. Fret ratio (F_{ECFP}/F_{EYFP}) of Epac1-cAMP probe with 1 μM ISO as a function of time from 0 to 30 min inside artificial cells. The data were expressed as mean ± standard deviation (SD), n = 3 independent replicates. Source data are provided as a Source Data file.

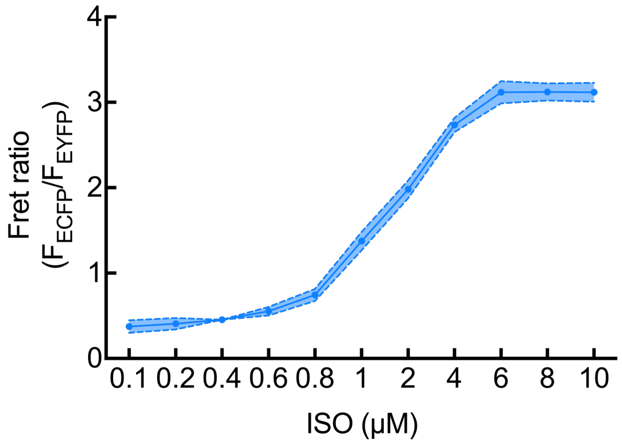

Supplementary Fig. 14. Fret ratio (F_{ECFP}/F_{EYFP}) of Epac1-cAMP with a variation of ISO concentration from 0.1 to 10.0 μM after 30 minutes inside artificial cells. The data were expressed as mean ± standard deviation (SD), n = 3 independent replicates. Source data are provided as a Source Data file.

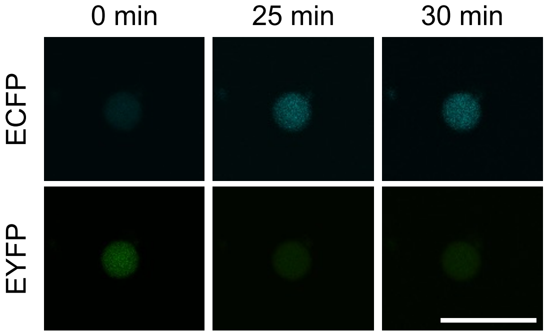

Supplementary Fig. 15. The representative time-series images of artificial cells (d=3.0 μm) stimulated by 1.0 μM ISO for the intracellular cAMP production at 0, 25, 30 min, with top row images taken by blue channel and bottom row images taken by green channel. The scale bar is 10.0 μm.

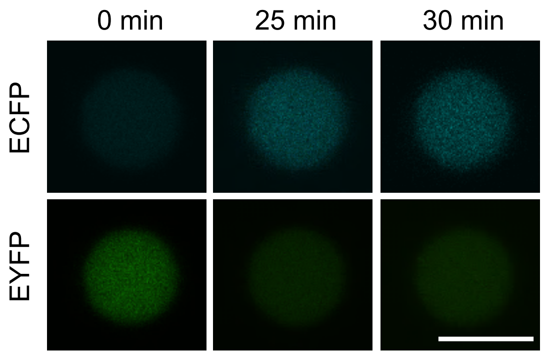

Supplementary Fig. 16. The representative time-series images of artificial cells (d=10.0 μm) stimulated by 1.0 μM ISO for the intracellular cAMP production at 0, 25, and 30 min, with top row images taken by blue channel and bottom row images taken by green channel. The scale bar is 10.0 μm.

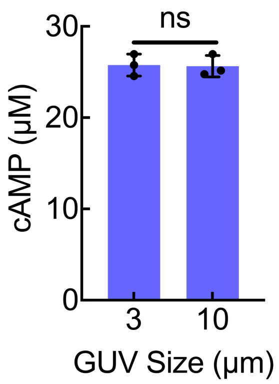

Supplementary Fig. 17. The cAMP concentration in artificial cells with diameter of 3.0 μm and 10.0 μm at 30 min with the addition of 1.0 μM ISO. The data were expressed as mean ± standard deviation (SD), n = 3 independent replicates. $^{ns}P > 0.05$, two-tailed t-test. Source data are provided as a Source Data file.

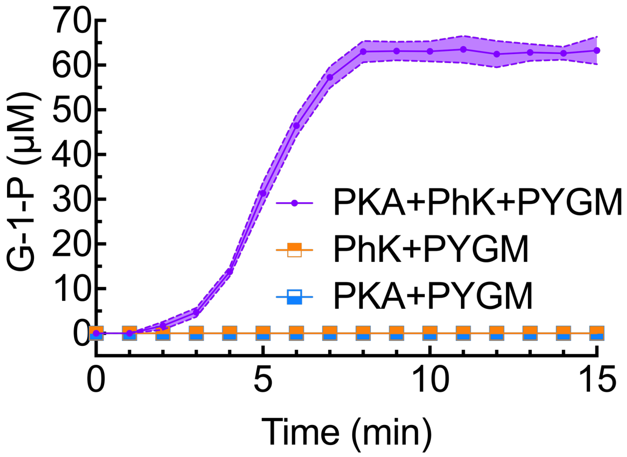

Supplementary Fig. 18. The concentration of produced G-1-P as a function of time inside artificial cells containing 1.0 µM cAMP, 0.8 mM ATP, 0.5 mM glycogen, 1.0 U/mL PKA, 1.4 U/mL PhK, 1.0 U/mL PYGM (purple curve), as well as the control groups without PKA (orange curve) or PhK (blue curve). The data were expressed as mean ± standard deviation (SD), n = 3 independent replicates. Source data are provided as a Source Data file.

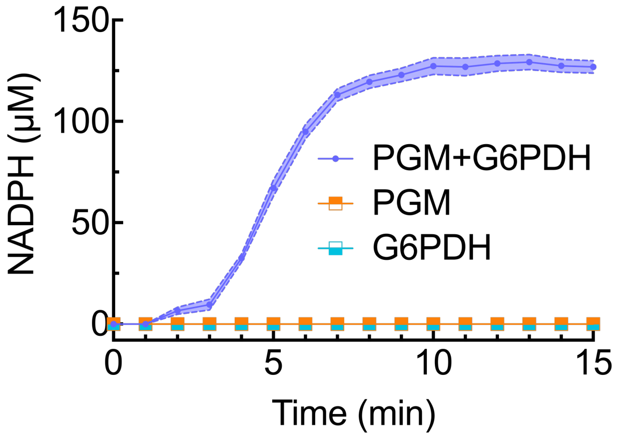

Supplementary Fig. 19. The concentration of produced NADPH as a function of time inside artificial cells containing 1.0 μM cAMP, 0.8 mM ATP, 0.5 mM glycogen, 0.2 mM $NADP^+$, 1.0 U/mL PKA, 1.4 U/mL PhK, 1.0 U/mL PYGM, 1.0 U/mL PGM, and 1.0 U/mL G6PDH (purple curve), as well as the control groups without G6PDH (orange curve) or PGM (blue curve). The data were expressed as mean ± standard deviation (SD), n = 3 independent replicates. Source data are provided as a Source Data file.

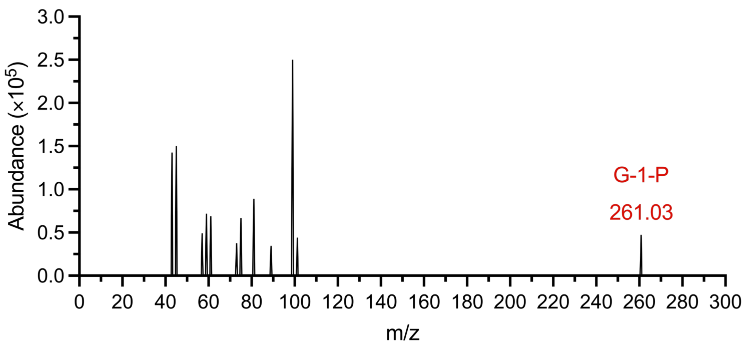

Supplementary Fig. 20. Mass spectrum of G-1-P in artificial cells. Source data are provided as a Source Data file.

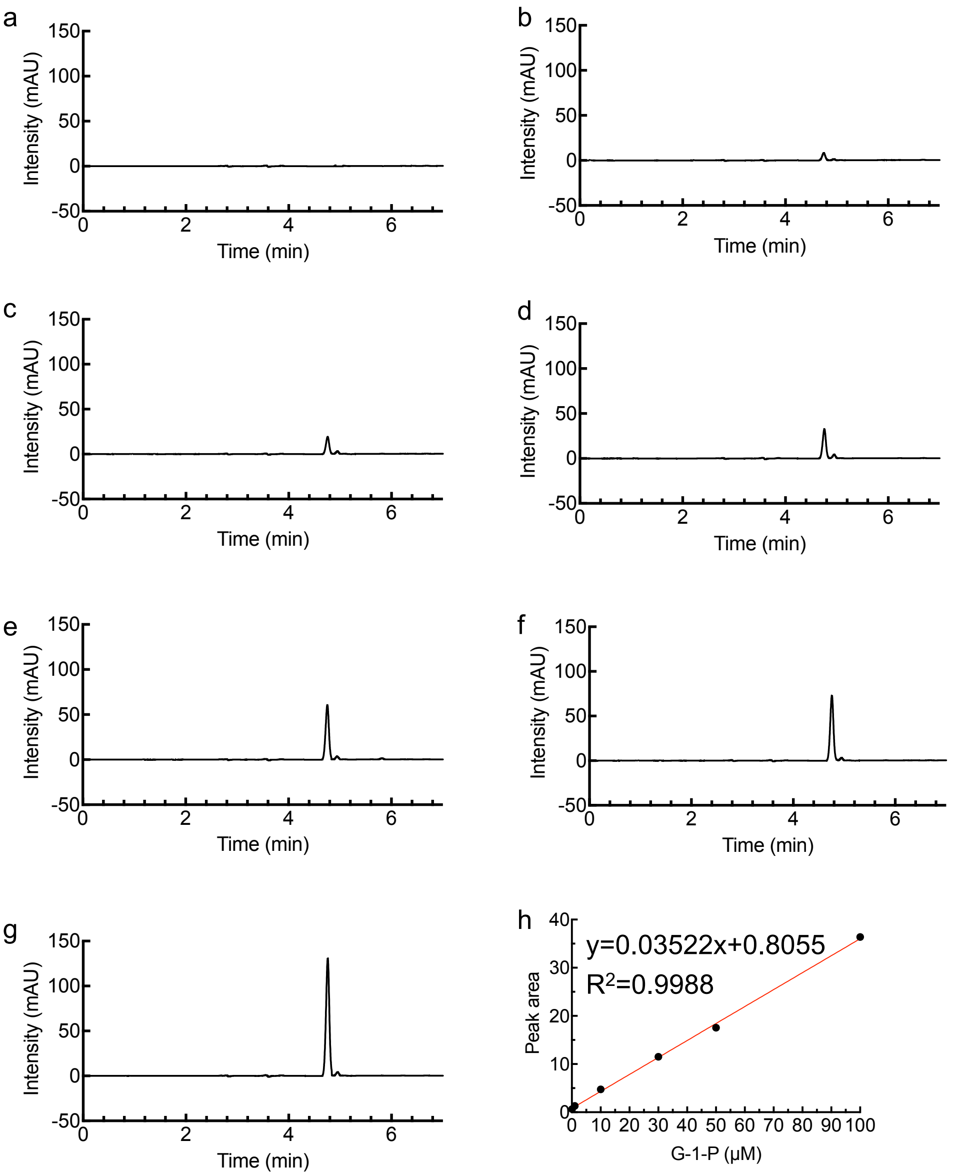

Supplementary Fig. 21. Representative high-performance liquid chromatography (HPLC) chromatograms of G-1-P at concentrations of 0 μM (a), 0.1 μM (b), 1 μM (c), 10 μM (d), 30 μM (e), 50 μM (f), and 100 μM (g). (h) The calibration curve of G-1-P detected by HPLC. Source data are provided as a Source Data file.

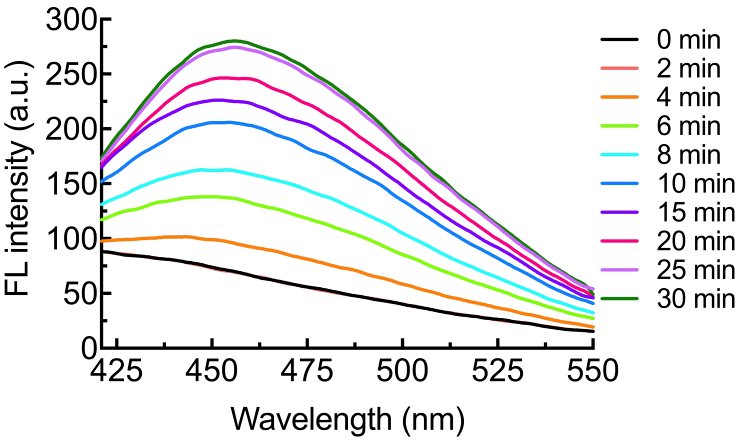

Supplementary Fig. 22. Fluorescence spectra of the produced NADPH inside artificial cells as a function of time with the stimulation of 1.0 μM ISO. Source data are provided as a Source Data file.

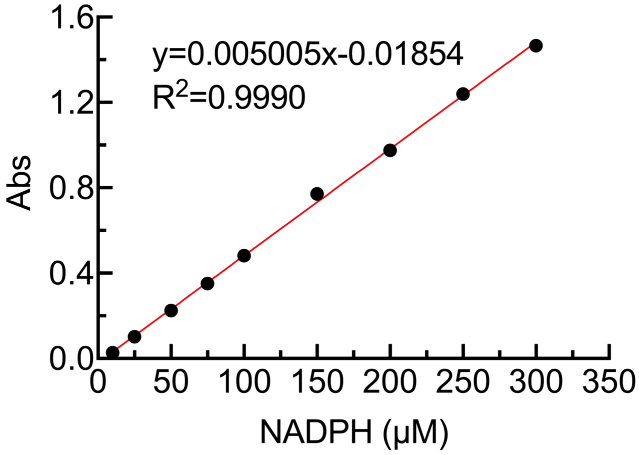

Supplementary Fig. 23. The calibration curve of NADPH within artificial cells.
Source data are provided as a Source Data file.
