# Supplementary material for: An artificial cell capable of signal transduction mediated by ADRB2 for the regulation of glycogenolysis
Source: Nat Commun. 2026 Jan 16;17:1795. doi: 10.1038/s41467-026-68503-3 (PMC12916806; doi:10.1038/s41467-026-68503-3)
Supplement: Supplementary file 3 — Description of Additional Supplementary Files [file 41467_2026_68503_MOESM3_ESM.pdf]

Description of Additional Supplementary Files

Supplementary Data 1: Sequences of gene of ADRB2, Gsα, ADCY5 and Epac1-cAMP used in current study.
