# Supplementary material for: An artificial cell capable of signal transduction mediated by ADRB2 for the regulation of glycogenolysis
Source: Nat Commun. 2026 Jan 16;17:1795. doi: 10.1038/s41467-026-68503-3 (PMC12916806; doi:10.1038/s41467-026-68503-3)
Supplement: Supplementary file 5 — Reporting Summary [file 41467_2026_68503_MOESM5_ESM.pdf]

nature portfolio

Corresponding author(s): Xiaojun Han

Last updated by author(s): 2025-12-18

Reporting Summary

Nature Portfolio wishes to improve the reproducibility of the work that we publish. This form provides structure for consistency and transparency in reporting. For further information on Nature Portfolio policies, see our Editorial Policies and the Editorial Policy Checklist.

Statistics

For all statistical analyses, confirm that the following items are present in the figure legend, table legend, main text, or Methods section.

n/a	Confirmed	
☐	☒	The exact sample size (n) for each experimental group/condition, given as a discrete number and unit of measurement
☐	☒	A statement on whether measurements were taken from distinct samples or whether the same sample was measured repeatedly
☐	☒	The statistical test(s) used AND whether they are one- or two-sided<br>*Only common tests should be described solely by name; describe more complex techniques in the Methods section.*
☒	☐	A description of all covariates tested
☒	☐	A description of any assumptions or corrections, such as tests of normality and adjustment for multiple comparisons
☐	☒	A full description of the statistical parameters including central tendency (e.g. means) or other basic estimates (e.g. regression coefficient) AND variation (e.g. standard deviation) or associated estimates of uncertainty (e.g. confidence intervals)
☐	☒	For null hypothesis testing, the test statistic (e.g. F, t, r) with confidence intervals, effect sizes, degrees of freedom and P value noted<br>*Give P values as exact values whenever suitable.*
☒	☐	For Bayesian analysis, information on the choice of priors and Markov chain Monte Carlo settings
☒	☐	For hierarchical and complex designs, identification of the appropriate level for tests and full reporting of outcomes
☒	☐	Estimates of effect sizes (e.g. Cohen's d, Pearson's r), indicating how they were calculated

Our web collection on statistics for biologists contains articles on many of the points above.

Software and code

Policy information about availability of computer code

Data collection	Laser scanning confocal microscope (Olympus FV 3000, Japan) and inverted fluorescence microscope (Olympus IX73, Japan) were used to obtain all fluorescence images. A microplate reader (Molecular Devices, SpectraMax iD3, Germany) was used to meansure the protein concentration. An ultrasonic homogenizer (Scientz, JY92-IIN, China) was used to lyse cells. A Cary 60 UV–vis spectrophotometer (Agilent, USA) was used for UV–vis spectra. A fluorescence spectrometry (PerkinElmer LS55, USA) was used for photoluminescence spectroscopy measurements. Amersham Imager 600 was used to capture the protein bands (GE HealthCare, USA). HPLC analysis was performed on an UltiMate 3000 HPLC system (Thermo Fisher Scientific, USA). Flow cytometry was performed on a flow cytometer (BD FACS Aria Fusion, USA). LC-MS was performed on a liquid chromatography-mass spectrometry (Agilent 1290Ⅱ-6545, USA). An automated cell counter (Countess II, Thermo Fisher Scientific, USA) was used to characterize the density of artificial cells.
Data analysis	All statistical analyses were performed using Origin 2024 and GraphPad Prism 10.0. The intensities of fluorescence images were analyzed using ImageJ 1.8.0.

For manuscripts utilizing custom algorithms or software that are central to the research but not yet described in published literature, software must be made available to editors and reviewers. We strongly encourage code deposition in a community repository (e.g. GitHub). See the Nature Portfolio guidelines for submitting code & software for further information.

All manuscripts must include a data availability statement. This statement should provide the following information, where applicable:

- Accession codes, unique identifiers, or web links for publicly available datasets
- A description of any restrictions on data availability
- For clinical datasets or third party data, please ensure that the statement adheres to our policy

Data

Policy information about availability of data

The source data underlying Fig. 2b, 2c, 2d, 2e, 2f, 2g, 2h, 2i, 2j, 2k, 2l, 3b, 3c, 3f, 3h, 3i, 4d, 4e, 4f, 4g, 4i, 4k, 4e, 5b, 5c, 5d, 5e, 5f, 5g, 5h, 6a, 6c, 6d, 6f, 6g and Supplementary Fig. 4, 5, 6b, 7b, 8, 9, 10, 11, 12, 13, 14, 17, 18, 19, 20, 21, 22, 23 are provided as a Source Data file.

Research involving human participants, their data, or biological material

Policy information about studies with human participants or human data. See also policy information about sex, gender (identity/presentation), and sexual orientation and race, ethnicity and racism.

Reporting on sex and gender	N/A
Reporting on race, ethnicity, or other socially relevant groupings	N/A
Population characteristics	N/A
Recruitment	N/A
Ethics oversight	N/A

Note that full information on the approval of the study protocol must also be provided in the manuscript.

Field-specific reporting

Please select the one below that is the best fit for your research. If you are not sure, read the appropriate sections before making your selection.

☒ Life sciences ☐ Behavioural & social sciences ☐ Ecological, evolutionary & environmental sciences

For a reference copy of the document with all sections, see nature.com/documents/nr-reporting-summary-flat.pdf

Life sciences study design

All studies must disclose on these points even when the disclosure is negative.

Sample size	The gel images of ADRB2, Gsα, ADCY5 and Epac1-cAMP were repeated three times. The size distribution of the artificial cells was determined by analyzing microscopy images of at least 300 individual GUVs. The reconsititution of ADRB2-Gsα was repeated three times. The reconsititution of ADCY5 was repeated three times. The co-reconsititution of ADRB2-Gsα and ADCY5 was repeated three times. The cAMP production in artificial cells was repeated three times. The western blots of ADRB2, PhK anfd PYGM were repeated three times. The NADPH production in artificial cells was repeated three times.
Data exclusions	No such exclusion in this paper.
Replication	The experiments were independently replicated at least three times.The reproducible results were always obtained.
Randomization	No randomization was used.
Blinding	No blinding was necessary for this study.

Reporting for specific materials, systems and methods

We require information from authors about some types of materials, experimental systems and methods used in many studies. Here, indicate whether each material, system or method listed is relevant to your study. If you are not sure if a list item applies to your research, read the appropriate section before selecting a response.

Materials & experimental systems

n/a	Involved in the study	
☐	☒	Antibodies
☐	☒	Eukaryotic cell lines
☒	☐	Palaeontology and archaeology
☒	☐	Animals and other organisms
☒	☐	Clinical data
☒	☐	Dual use research of concern
☒	☐	Plants

Methods

n/a	Involved in the study	
☒	☐	ChIP-seq
☐	☒	Flow cytometry
☒	☐	MRI-based neuroimaging

Antibodies

Antibodies used

Rabbit anti-PhKA2 polyclonal antibody (24658-1-AP), rabbit anti-PYGM-Specific polyclonal antibody (19716-1-AP) and ADRB2 polyclonal antibody (29864-1-AP) were purchased from Proteintech (China). Anti-PYGL (phospho S430) + PYGM (phospho S430) antibody (EPR20852-26) were purchased from Abcam (China). Rabbit anti-phospho-Serine polyclonal antibody (bs-11993R) and Rabbit GAPDH polyclonal antibody (bs-10900R) were purchased from Bioss (China). HRP-labeled goat anti-rabbit IgG (H+L) (A0208) were purchased from Beyotime (China).

Validation

1.Rabbit anti-PhKA2 polyclonal antibody (Proteintech, No. 24658-1-AP)
https://www.ptgcn.com/products/PHKA2-Antibody-24658-1-AP.htm#tested-applications
2.Rabbit anti-PYGM-Specific polyclonal antibody (Proteintech, No. 19716-1-AP)
https://www.ptgcn.com/products/PYGM-Specific-Antibody-19716-1-AP.htm#tested-applications
3. ADRB2 polyclonal antibody (Proteintech, No. 29864-1-AP)
https://www.ptgcn.com/products/ADRB2-Antibody-29864-1-AP.htm#tested-applications
4. Anti-PYGL (phospho S430) + PYGM (phospho S430) polyclonal antibody (Ancam, No. ab314428)
https://www.abcam.cn/products/primary-antibodies/pygl-phospho-s430-pygm-phospho-s430-antibody-epr20852-26-ab314428
5. Rabbit anti-phospho-serine polyclonal antibody (Bioss, No.bs-11993R)
https://www.biosschina.com/productDetail?goods_id=15900
6. Rabbit GAPDH polyclonal antibody (Bioss, No. bs-10900R)
https://www.biosschina.com/productDetail?goods_id=22648

Eukaryotic cell lines

Policy information about cell lines and Sex and Gender in Research

Cell line source(s)

Sf9 cell line was purchased from Procell Life Science & Technology (China).

Authentication

Sf9 cell line was authenticated through multiplex amplification for species identification by Procell Life Science & Technology (China).

Mycoplasma contamination

Sf9 cell line were tested negative for mycoplasma contamination.

Commonly misidentified lines
(See ICLAC register)

N/A

Plants

Seed stocks

N/A

Novel plant genotypes

N/A

Authentication

N/A

Flow Cytometry

Plots

Confirm that:

☒ The axis labels state the marker and fluorochrome used (e.g. CD4-FITC).

☒ The axis scales are clearly visible. Include numbers along axes only for bottom left plot of group (a 'group' is an analysis of identical markers).

☒ All plots are contour plots with outliers or pseudocolor plots.

☒ A numerical value for number of cells or percentage (with statistics) is provided.

Methodology

Sample preparation

GUVs containing membrane proteins were obtained and were adjusted to the density of 10000000 per mL.

Instrument

Flow cytometry was performed on a flow cytometer (BD FACS Aria Fusion, USA).

Software

Flow cytometry data was analysed using Flowjo 10.8.1 software.

Cell population abundance

The density of GUVs was 10000000 per mL. The density of artificial cells was directly quantified using an automated cell [illegible]

counter, by adding GUV solution (10 µL) to the cell counting chamber.

Gating strategy

The threshold was adjusted based on forward scatter (FSC) and side scatter (SSC) to exclude small lipid aggregates, with only events with FSC-A >10^4 and SSC-A >10^3 to be GUVs. The flow cytometry data acquisition for each sample included 104 GUVs. GUVs without membrane proteins were analyzed to establish the autofluorescence background with a threshold to be 99% of this population.

☐ Tick this box to confirm that a figure exemplifying the gating strategy is provided in the Supplementary Information.
